# Accuracy and quality of massively parallel DNA pyrosequencing

**DOI:** 10.1186/gb-2007-8-7-r143

**Published:** 2007-07-20

**Authors:** Susan M Huse, Julie A Huber, Hilary G Morrison, Mitchell L Sogin, David Mark Welch

**Affiliations:** 1 Josephine Bay Paul Center, Marine Biological Laboratory at Woods Hole, MBL Street, Woods Hole, MA 02543, USA

## Abstract

**Background:**

Massively parallel pyrosequencing systems have increased the efficiency of DNA sequencing, although the published per-base accuracy of a Roche GS20 is only 96%. In genome projects, highly redundant consensus assemblies can compensate for sequencing errors. In contrast, studies of microbial diversity that catalogue differences between PCR amplicons of ribosomal RNA genes (rDNA) or other conserved gene families cannot take advantage of consensus assemblies to detect and minimize incorrect base calls.

**Results:**

We performed an empirical study of the per-base error rate for the Roche GS20 system using sequences of the V6 hypervariable region from cloned microbial ribosomal DNA (tag sequencing). We calculated a 99.5% accuracy rate in unassembled sequences, and identified several factors that can be used to remove a small percentage of low-quality reads, improving the accuracy to 99.75% or better.

**Conclusion:**

By using objective criteria to eliminate low quality data, the quality of individual GS20 sequence reads in molecular ecological applications can surpass the accuracy of traditional capillary methods.

## Abstract 2

Error rates were estimated for the Roche GS20 massively parallel pyrosequencing system, and several factors were identified that can be used to remove low-quality reads, improving the accuracy to 99.75% or better.

## Background

Direct interrogation of microbial genomes based upon comparisons of orthologous gene sequences or metagenomic surveys provides a means to assess the diversity of microbial communities without requiring the cultivation of microbes in the laboratory. Since the cost of cloning DNA templates and capillary-based DNA sequencing constrains the number of sequences included in most of these investigations, the detection of low abundance taxa demands surveys that are many orders of magnitude larger than those reported in the literature. Massively parallel pyrosequencing on the Roche GS20 system developed by 454 Life Sciences offers a means to more extensively sample molecular diversity in microbial populations. It is now possible to generate hundreds of thousands of short (100-200 nucleotide) DNA sequence reads in a few hours without requiring the preparation of sequence templates by conventional cloning. In the near future, technical advances will likely increase the number and length of sequence reads.

Pyrosequencing technology relies upon enzyme cascades and CCD luminescence detection capabilities to measure the release of inorganic pyrophosphate with every nucleotide incorporation [1]. The GS20 system takes advantage of DNA capture beads that contain, on average, one single-stranded template, which is amplified to millions of copies in an oil emulsion PCR (emPCR). The beads are then distributed on a solid-phase sequencing substrate (a PicoTiterPlate™) with 1.6 million wells that can each hold a bead and additional reagents, including polymerase, luciferase, and ATP sulfurylase. Microfluidics cycle each of the four nucleotide triphosphates over the PicoTiterPlate™, and incorporation of a nucleotide releases pyrophosphate, the substrate for a luminescence reaction, which is recorded with a cooled CCD camera. The record of intensity of each flow of a nucleotide is a flowgram, analogous to a chromatogram that reports the order of A, C, G and T residues from a DNA sequencing template. Flowgram values correspond to the homopolymer length for that base. The average number of wells with detectable sequencing templates is about 450,000, which produces about 200,000 usable reads. This new methodology brings with it different sources of error to traditional dideoxy capillary sequencing. Since the nucleotide triphosphates are flowed one at a time, substitutions are less likely than with traditional methods. However, it is sometimes difficult to resolve the intensity of luminescence produced when a homopolymer is encountered. The result can be ambiguity of homopolymer length, particularly for longer homopolymers. In addition, insufficient flushing between flows can cause single base insertions (carry forward events) usually near but not adjacent to homopolymers. Insufficient nucleotides within a flow can cause incomplete extension within homopolymers. Generally, an excess of intermediate flowgram values indicates a poor quality read [2]. The GS20 software makes corrections for carry forward and incomplete extensions (CAFIE); it shortens reads from the 3' end until fewer than 3% of the remaining flowgram values are of intermediate value, and it removes reads if the trimming falls below a threshold length. The software identifies as ambiguous flow cycles in which no flowgram value was greater than 0.5. If 5% or more of the flow cycles for a read are ambiguous, the read is removed.

The assembly of many overlapping pyrosequencing reads can produce highly accurate consensus sequences [3,4]. Wicker *et al*. [5] compared assemblies of the barley genome produced by reads from GS20 pyrosequencing and from ABI dideoxy sequencing. Both methods produced consensus sequences with error rates of approximately 0.07% at each consensus position. Gharizadeh *et al*. [6] compared pyrosequences with Sanger dideoxy methods for 4,747 templates. Comparisons of the traditional capillary sequences with the 25-30 nucleotide pyrosequence reads demonstrated similar levels of read accuracy. Assemblies of massively parallel pyrosequencing reads of plastid genomes from *Nandina *and *Platanus *exhibited overall error rates of 0.043% and 0.031%, respectively, in the consensus sequence [4]. The generation of consensus sequences to improve accuracy, however, is generally not appropriate for studies that seek information about natural variation from every read. For example, in metagenomic [7] or PCR amplicon [8] libraries from environmental DNA samples, each sequence read can theoretically represent DNA from a distinct gene from a complex mixture of microbial genes.

A viable but imperfect alternative to building consensus sequences for metagenomic and diversity investigations is to identify and remove pyrosequencing reads that are likely to be incorrect. For example, Gilbert *et al*. [9], in a study of ancient woolly mammoth mitochondrial DNA, removed pyrosequencing reads that were not 98% identical to previously sequenced mammoth mitochondrial DNA sequences, assuming that they must be poor quality. Dostie *et al*. [10] sequenced an amplicon library and discarded reads in which the PCR primer was not recognized by BLAST. These studies removed 15% and 7% of their reads, respectively, but it is not clear that these statistics improved the quality of the remaining data.

To explore error modalities, we used the GS20 system to generate more than 340,000 reads from a PCR amplicon library that was prepared from a collection of 43 reference templates of known sequence. Each reference template contains a distinct ribosomal RNA gene (rDNA), including the V6 hypervariable region from a collection of 43 divergent bacteria [11]. Differences between pyrosequences and their cognate reference sequences identified signatures of low quality data.

## Results

### Read accuracy

We obtained 340,150 reads that passed the GS20 quality filters, that is, flowgrams for each read: contained the correct base key at the start (a portion of the 454 primer used to differentiate reads from internal quality control sequences); included at least 84 flows; had fewer than 5% of flow cycles resulting in an ambiguous base call (N); and had fewer than 3% of flowgram values between 0.5 and 0.7 [12]. We aligned each read to its reference sequence using an optimized Needleman-Wunsch algorithm. Our data included 159,981 total errors over 32,801,429 bases. The error rate, defined as the number of errors (miscalled bases plus inserted and deleted bases) divided by the total number of expected bases, was 0.49%. As shown in Table 1, 39% of these errors correspond to homopolymer effects, including extension (insertions), incomplete extensions (deletions) and carry forward errors (insertions and substitutions). Carry forward occurs when an incomplete flush of base flow results in a premature incorporation of a base. The presence of homopolymers tends to increase the likelihood of both carry forward and incomplete extension with the GS20 sequencer [12]. Insertions were the most common type of error (36% of errors) followed by deletions (27%), ambiguous bases, Ns (21%), and substitutions (16%). It should be noted that the V6 region does not contain long or frequent homopolymers. The errors did not correlate significantly with distance along the sequence (R^2 ^< 0.03), indicating that, for the retained reads, there was not significant degradation of sequencing quality as the run progressed. Similar to the results of Margulies *et al*. [2], the error rate for test fragments (sequences used for GS20 diagnostics and included in all runs as part of the reagent stream), was much lower than for the reads, 0.1% bases in error in our experiment.

**Table 1:** Types of error

Error type	Number of occurrences	Percent of errors	Error rate
Insertions	58,337	36%	0.18%
Homopolymer extension and CAFIE	32,858	20%	0.10%
Not associated with homopolymers	25,479	16%	0.08%
Deletions	43,107	27%	0.13%
Incomplete homopolymer extension	13,868	9%	0.04%
Not associated with homopolymers	29,239	18%	0.09%
Mismatches	25,281	16%	0.08%
Homopolymer extension and CAFIE	16,725	10%	0.05%
Not associated with homopolymers	8,556	5%	0.03%
Ambiguous base calls (N)	34,184	21%	0.10%

Read errors	Number of occurrences	Cumulative percent of reads	Percent of reads

Reads with no errors (perfect match)	279,468	82%	82%
Reads with no more than one error	35,813	93%	11%
Reads with no more than two errors	11,651	96%	3%
Reads with more than two errors	13,218	100%	4%

Errors were classified as insertions, deletions, mismatches (substitutions) and ambiguous base calls (Ns). We further classified insertions, deletions and mismatches by their association with homopolymer effects. Deletions corresponding to an adjacent base are considered incomplete extension. Insertions and mismatches of the same base as an adjacent base are extensions. Insertions and mismatches that are the same as an upcoming homopolymer with no more than two intervening bases are considered carry forward errors.

Two transition mismatches, A to G and T to C, were more frequent than other mismatches, but the reverse transitions, G to A and C to T, were not. Nearly 70% of the homopolymer extensions were A/T. After correcting for the relative number of homopolymers of each type (A/T versus C/G) in the reference sequences (45% and 55%, respectively), the prevalence of A/T extensions was 24% higher than expected, and C/G was concomitantly lower. Although substantially less common, the carry-forward errors, where a base is inserted or substituted ahead of a homopolymer run of the same base (for example, GACTGGG could become GACGTGGG with a carry forward insertion of a G) reflected the same relative increase of A/T over C/G.

### Read quality

**Figure 1:**
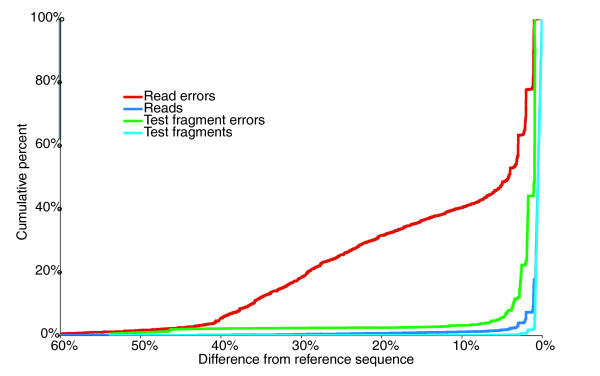
Low quality reads contribute disproportionately to the overall error rate. The graph shows the proportion of reads and test fragments at each percent difference from their reference sequence (individual error rate) and the proportion of errors contributed by reads and test fragments at each given difference (cumulative error rate). The vast majority of both experimental and test fragment reads contain few or no errors; only 5% of all reads and 0.6% of test fragments differ from their reference sequence by 2% or more. The experimental reads that have errors, however, are likely to have a large number of errors and thus be quite different from their reference sequence. For instance, 40% of all errors are from the 1% reads differing by at least 10% from their reference sequence. The GS20 test fragments, by contrast, show far fewer very low-quality sequences: only 3% of the test fragment errors are from sequences at least 10% different from their reference.

While the errors were evenly distributed along the length of the reference sequences, they were not evenly distributed among reads. Of the reads, 82% had no errors, 93% had no more than a single error, and 96% had no more than 2 errors. Conversely, a small number of reads, fewer than 2%, contained a disproportionate number of errors that account for nearly 50% of the miscalls for the entire dataset (Figure 1). We identified several diagnostic features that correlate well with the presence of a large number of errors in a read. These include average quality, length, and the presence of ambiguous base calls.

**Figure 2:**
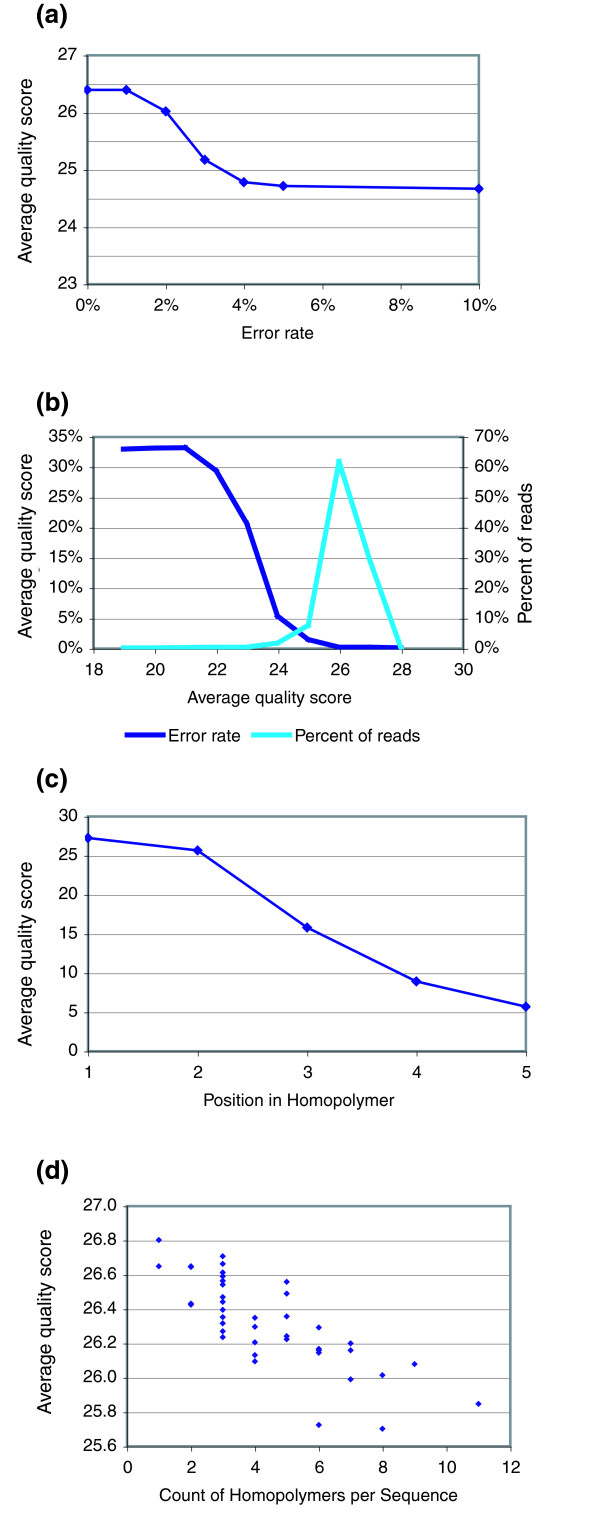
Quality scores are of limited use in predicting accuracy of unknown sequences. The quality scores reported by the GS20 software correlate with decreased confidence in calling the correct homopolymer length rather than the accuracy of the called bases. **(a, b) **The average quality score of reads decreases as the number of errors in the read increases. **(c) **The average quality score as a function of position in the homopolymer: as the length of the homopolymer increases, the quality scores decrease, for both correctly and incorrectly called bases. **(d) **The average quality scores of perfect reads containing differing numbers of homopolymers. The average quality scores decrease with the number of homopolymers. Our sequences contain only short homopolymers, primarily 3-mers. As the length and frequency of homopolymers increases, the expected quality scores will decrease. Without *a priori *knowledge of the number and length of homopolymers in a particular read, it will be difficult to assess an appropriate quality threshold - a low threshold may not cull data adequately and a high threshold may remove homopolymeric regions.

The output from the GS20 includes a quality score for every position in a read, and we found that the average quality score of a read is inversely proportional to the number of errors in that read (Figure 2a). In our data, reads with an average quality score above 25 had very few errors, but the number of errors per sequence was noticeably higher for any read where the average of quality scores fell below 25 (Figure 2b). However, the quality score of a position is not a measure of the confidence that the correct base is called at that position, as with a traditional PHRED score [13]. Instead, the GS20 quality score is a measure of confidence that the homopolymer length at that position is correct [2]. The quality scores drop as the homopolymer length increases, even when those homopolymers are correctly called (Figure 2c). Thus, as the number and length of homopolymers in a sequence increase, the average quality score decreases regardless of error (Figure 2d). This is evident even for V6 regions of rDNAs that contain relatively few and short homopolymers. The range of average quality scores is very narrow, from 24.7 to 26.4, compared to the range of quality scores for longer homopolymers, which range from 5 to 26. Predicting an average quality cutoff may not be feasible with data of mixed or unknown homopolymer lengths and frequencies.

**Figure 3:**
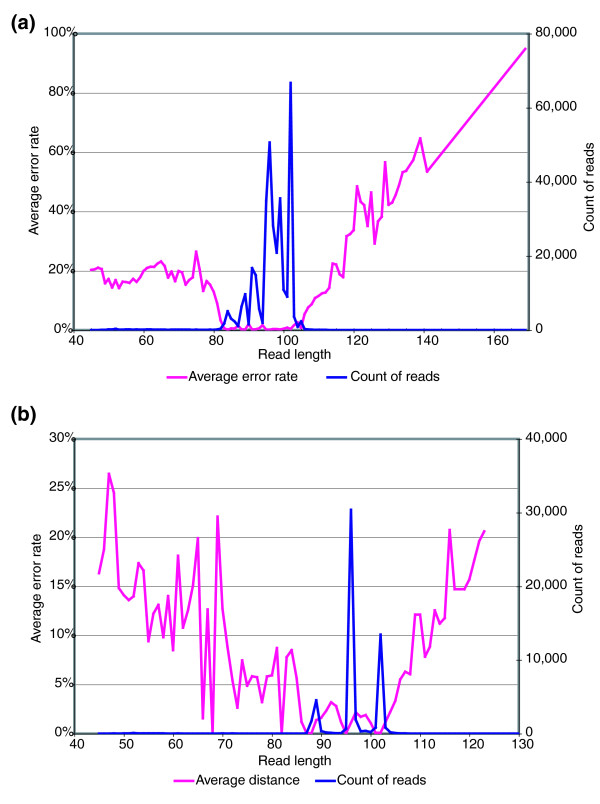
Error rates increase as read length diverges from predicted. The graphs show the average difference from the reference sequence for all reads of a given length, and the distribution of read lengths for all reads. The majority of reads peak at a few specific lengths. The number of reads beyond the peaks shown are too few to appear on the graph; however, they contain many more errors than the reads of the majority length(s). Perfect reads peak at only a few specific lengths. Sequences that fall outside of these lengths are unlikely to be truncated sequences or to have sequenced beyond the end of the primer. Instead they tend to be low-quality reads of spurious sequences. **(a) **The average error rate of sequences at each length for 56,700 reads of reference sequence 517. **(b) **The average error rate of sequences at each length for all reads combined. Even with a mixture of sequence lengths, the reads outside of the peak lengths are highly error prone.

Reads that are much shorter or much longer than their predicted length based on the GS20 flow order also had a very large proportion of errors, while those within a few bases of the expected length had very few errors (Figure 3). We compared read length and average error rate for each of the reference sequences and found that the distribution of high-quality reads peaked at the optimal length as well as a few common shorter lengths, while reads not associated with these clear peaks have much larger average error rates.

**Figure 4:**
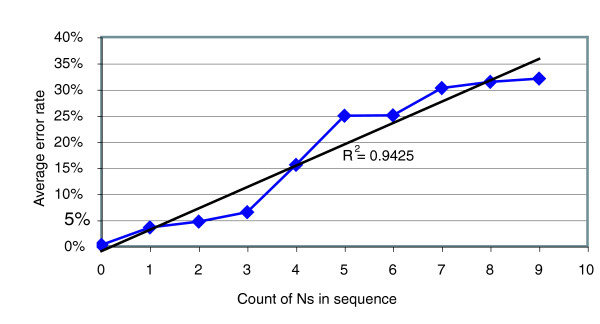
The presence of Ns correlates well with sequencing errors. The presence of an N in the sequence indicates the GS20's inability to accurately call a base at that position within the sequence. The number of other sequencing errors (substitutions, insertions and deletions) within a sequence read correlates with number of uncalled bases. By removing all reads that contain one or more Ns, the overall sequencing error rate drops substantially.

We observed a strong correlation between the presence of ambiguous base calls and other errors in a read (Figure 4). The presence of even a single ambiguous base in a read correlates strongly with the presence of other errors. Ambiguous base calls result from a failure of the GS20 system to identify any base at an individual position throughout an entire flow cycle. The GS20 bioinformatics pipeline removes reads having more than 5% of the flows resulting in an N; however, this can still result in reads with as many as 9 ambiguous bases calls, and many thousands with 1-2 ambiguous calls. We found that a more stringent threshold than that incorporated into the quality filtering software can substantially improve the quality. After excluding all reads containing ambiguous bases, the error rate is 0.24%, whereas the error rate of reads containing one or more Ns is 4.7% (*n *= 19,223, 5.6% of all reads) accounting for 54% of the errors. We reexamined the data from each of the two runs separately and found that although their initial error rates did differ slightly (0.58% versus 0.32%), they were essentially the same after removal of reads containing Ns (0.23% and 0.27%, respectively).

We compared error rates in the proximal primer region with error rates in the sequence following the primer. We found a positive, non-linear correlation between the two error rates (not shown). Primers with three or more errors are indicative of low-quality sequences downstream of the primer, but primers with fewer errors are not a good predictor of the overall error of the read. In our experiment, 3% of the reads contained inexact matches to the proximal primer, but removing them only reduced the error rate from 0.49% to 0.45%: most of this improvement reflects elimination of incorrect base calls in the primer sequence. When all reads with Ns, primer errors, and long or short read lengths were removed the total error rate was reduced to 0.16%. The remaining errors correspond to insertions (51%), deletions (33%) and substitutions (16%).

We looked for spatial patterns of read errors using the xy location of each read on the plate (available in the GS20 output files). There can be some minor edge effects but these are not consistent across runs and do not represent a large enough portion of the reads under normal conditions to be a useful tool. However, we note that the total number of errors due to edge effects will be greater in experiments that use 4 or 16 lane gaskets, as the total number of edges will be proportionally larger.

## Discussion

The massively parallel pyrosequencing of emulsification PCR-based templates holds great promise for revolutionizing high-throughput sequencing. However, there is concern over the potentially high degree of error, particularly for applications that cannot rely on consensus of large assemblies. Consensus-based projects would also benefit from a lower error rate, as fewer sequences would be required to build a reliable consensus. The original description of the 454 Life Sciences system reported an error rate for shotgun library reads of four bases per hundred nucleotide positions. The test fragment data in that publication had much lower error rates despite the fact that the test fragments have extensive homopolymers and are designed to be difficult to read correctly. This discrepancy indicates that the basic method of pyrosequencing, luminescence detection and flow intensity resolution, is sound, and suggests that the higher error with experimental data may come from the experimental manipulation of the sequences prior to pyrosequencing. Margulies *et al*. [2] suggested that this may be due to multiple sequences binding to an individual bead prior to the emPCR amplification, resulting in a heterogeneous amplification pool. The GS20 quality filters will eliminate sequences from beads that contain two highly divergent DNA templates but the software will attempt to interpret flowgrams from a single bead that contains two similar but non-identical sequences. Unlike shotgun genomic data, V6-tag data may have large numbers of highly similar sequences. It is, therefore, even more important in V6-tag and metagenomic sequencing to remove reads that may result from multi-templated beads.

We conducted an in-depth analysis of experimentally generated GS20 reads by sequencing an amplicon library made from a set of clones of known sequence. We found an error rate (incorrect bases/total number of expected nucleotides) of 0.49%, considerably lower than that reported by Marguiles *et al*. [2] but still higher than they or we found for test fragment data. Significantly, we found that the errors in our experimental reads were not randomly distributed across all reads: 86% of the reads contain no errors, while reads that differ from the reference sequence by more than 4% contained nearly 50% of the errors (Figure 1). In contrast, errors were much more randomly distributed in our test fragments, where 50% of the errors were from those fragments that differ by less than 1% from the reference. A multi-templated bead would frequently have multiple bases at a position, which could cause indeterminate flows - neither base having ample luminescence to clearly register. The convergence of the error rates of the two separate sequencing runs when the reads containing Ns were removed is consistent with the multi-templated bases as the primary source of error. The error distribution across reads and the similarity of error rates for reads with no Ns are consistent with a high general accuracy of the pyrosequencing method and poor resolution of a small number of beads with a heterogeneous amplification population.

If heterogeneous templates on a single bead represented a major contribution to observed errors in a low quality read, we anticipate a disproportionate number of errors would occur in sequences that correspond to low abundance templates in the original emPCR reactions; if a low-frequency strand shares a bead with another sequence, the other sequence is likely to be different. In contrast, high-frequency sequences are more likely to be contaminated by an identical sequence. Our data match this pattern. The removal of the bulk of the errors via the removal of reads with ambiguous bases is also consistent with multi-templated beads. All of our reads shared the same proximal primer, and would, therefore, sequence with few errors in the primer, even on multi-templated beads. Other experiments with heterogeneous primers may find primer fidelity to be more useful at identifying low-quality reads.

A significant decrease in the heterogeneous amplification population (HAP) between the original reported experiment and ours is likely given the improvements in the protocol developed by 454 Life Sciences. Unfortunately, it suggests that the highest single source of error in an emPCR-based pyrosequencing experiment may vary from experiment to experiment. The evidence from our two separate sequencing runs, however, is that the removal of reads with Ns is a good surrogate for the removal of reads from multi-templated beads. Nonsynchronized extension of fragments will also produce Ns. These will also be culled when reads with Ns are removed. Advances in pyrosequencing that would reduce the occurrence of multi-templated beads, reduce nonsynchronized extension, or better identify these errors in the base-calling software could significantly improve the overall accuracy of the technology.

Our analysis of the distribution of the types of error in pyrosequencing of emPCR libraries suggests ways of identifying and removing these HAP-hazards: reads with a disproportionately large number of errors are disproportionately likely to contain ambiguous bases (Ns) and to be aberrantly long or short. Short reads arise from short fragments on emPCR beads, but also, and perhaps more likely, from sequential deletion of a read by the software in the GS20 machine, which successively trims bases presumed to be in error from the end of reads. The more bases trimmed, the more likely the entire read is of dubious quality. These errors may be multi-templated beads of similar sequence, or nonsynchronized extension of the templates. Once fragments lose synchrony, they will have successively more errors as the read extends. A reduced threshold for removing reads that have bases trimmed from the end might remove many of these poor-quality short reads. Many sequencing projects cannot judge an appropriate read length, although it is always possible to detect and remove short reads. Reads of aberrant length represented only a small fraction, approximately 1%, of our data, and most of these reads, >60%, also contain Ns.

## Conclusion

Our analysis of the GS20 sequencing error rate of the V6 region of bacterial rRNA genes shows a marked improvement over the original error rates published by Margulies *et al *[2]. The largest source of errors may be due to multi-templated beads, and enhancements to both the chemistry protocol for the GS20 and the built-in bioinformatics software may account for the change in error rates. Our results highlight that a small proportion of low quality reads, presumably from multi-templated beads, are responsible for the majority of sequencing errors. The ability to identify and remove these reads is the best way to improve the accuracy of the entire dataset. It is not a replacement for assigning quality scores to detect the position of miscalled bases. The interpretation of chromatograms by programs such as PHRED [13] employs quality scores that reflect the probability of any type of base call error. Although it uses the same scale, the GS20 software generates quality values based on the probability of homopolymer extension rather than probability of a correct base call.

Regardless of the ultimate cause of poor reads, the presence of even a single ambiguous base (N) was an effective indicator of low-quality sequence. The removal of all reads containing one or more Ns can drastically improve the overall quality of the remaining dataset, reducing the error rate from about 0.5% to about 0.25% (Table 2). For our data, this strategy eliminated only 6% of the total reads. By excluding approximately 1% of all reads whose lengths lie outside of the main distribution, as well as those with inexact matches to the primer, the error rate for the V6-tag data dropped to less than 0.2%. The pyrosequencing technology provides such a large number of reads that the elimination of even 10% or more of the reads in a data set should be more than offset by the increase in quality of the remaining reads.

**Table 2:** Identifying low-quality reads and their contribution to the error rate

Data selection	Percent of reads	Error rate
All reads	100.0%	0.49%
Reads with no Ns	94.4%	0.24%
Reads with one or more Ns	5.6%	4.7%
Reads with length ≥81 and ≤108	98.8%	0.33%
Reads with length <81 or >108	1.2%	18.9%
Reads with no Ns and length ≥81 and ≤108	93.3%	0.20%
Reads with no proximal errors	97.0%	0.45%
Reads with fewer than three proximal errors	>99.99%	0.48%
Reads with more than three proximal errors	<0.01%	12.2%
Reads with no Ns and length ≥81 and ≤108 and no proximal errors	90.6%	0.16%

Removing reads with Ns is the most effective means we found of removing low-quality data and improving the error rates. Read lengths that are either longer or shorter than expected, and are outside the peak of common reads, also correlate strongly with incorrect reads.

Our strategy for detecting low quality reads circumvents the need to generate consensus sequences for improving data quality in massively parallel pyrosequencing experiments of environmental DNA. Our criteria for detecting reads with errors allows for the acquisition of pyrosequencing data in the context of molecular ecology that can surpass the accuracy of traditional capillary methods.

## Materials and methods

### Generation of 1 kb clone library and selection of clones for pyrosequencing

DNA was extracted according to Huber *et al*. [14] from diffuse flow hydrothermal vent samples as described in Sogin *et al*. [8]. PCR primers were designed using ARB software [15] to target the bacterial 16S rDNA. The primers used were 337 F (5' CAN CCT ACG GGN GGC NGC) and 1391R (5' GAC GGG CGG TGW GTN CA). The amplification mix contained 5 units *Pfu *Turbo polymerase (Stratagene, La Jolla, CA, USA), 1× *Pfu *reaction buffer, 200 μM dNTPs (Pierce Nucleic Acid Technologies, Milwaukee, WI, USA), and 0.2 μM each primer in a volume of 100 μl. Environmental DNA (3-10 ng) was added to 3 separate 30 μl amplification mixes. A positive control (*Marinobacter aquaeolei *genomic DNA) and two negative controls (no DNA and genomic DNA from the archaeon *Methanococcus jannaschii*) were also run. An initial denaturation step of 3 min at 94°C was followed by 30 cycles of 94°C for 30 s, 55°C for 45 s, and 72°C for 2 minutes. The final extension step was 72°C for 2 minutes. Following PCR, three reactions for each sample were combined, purified, and concentrated using the MinElute PCR Purification Kit (Qiagen, Valencia, CA, USA) according to the manufacturer's instructions. PCR product quality was assessed on a 0.8% agarose gel stained with ethidium bromide and ligated with pCR4-TOPO vector for 20 minutes at room temperature and transformed with TOP10 electrocompetent cells according to the manufacturer's instructions (Invitrogen, Carlsbad, CA, USA). Colonies for each library were randomly selected and grown in SuperBroth with 50 mg/ml kanamycin in 96 deep-well blocks overnight. Alkaline lysis template preparation was carried out on cell pellets using the RevPrep Orbit II (Genomic Solutions, Ann Arbor, MI, USA) or the Biotech RoboPrep2500 (MWG Biotech, Ebersberg, Germany). The 1,000 base-pair amplicons were sequenced bidirectionly using primers T3 (5'-ATT AAC CCT CAC TAA AGG GA) and T7 (5'-TAA TAC GAC TCA CTA TAG GG), and on an ABI 3730 × l genetic analyzer. Sequences were aligned with MUSCLE (with parameters -diags and -maxiters 10) [16] and manually manipulated in the BioEdit 7.0.1 program [17]. Distance matrixes were calculated using quickdist [8], and taxonomic identities determined using RDP-II Classifier [18]. Sequences were trimmed to include only the V6 region of the gene, the distance matrix re-calculated, and from this analysis, 43 divergent sequences were chosen for further experimentation. The average length of the V6 region for these clones was 101 bases, ranging from 95 to 109, with one longer reference of 183 bases. The 16S rDNA sequences are deposited at GenBank under accession numbers DQ909092, DQ909128DQ909132, DQ909133, DQ909142, DQ909144, DQ909158, DQ909184, DQ909202, DQ909204, DQ909218, DQ909223, DQ909224, DQ909248, DQ909251, DQ909253, DQ909266, DQ909274, DQ909337, DQ909368. DQ909392, DQ909396, DQ909400, DQ909414, DQ909423, DQ909438, DQ909440, DQ909465, DQ909474, DQ909498, DQ909513, DQ909519, DQ909538, DQ909603, DQ909618, DQ909631, DQ909662, DQ909688, DQ909702, DQ909706, DQ909719. DQ909727, DQ909753.

### Generation of known V6 amplicon library

We treated each plasmid with plasmid-safe DNAase (Epicentre, Madison, WI, USA) to remove *Escherichia coli *genomic DNA and confirmed that each plasmid produced an amplification product of the expected size with primers targeting the V6 region of the bacterial rDNA according to Sogin *et al*. [8]. We then pooled the individual plasmids and amplified with the primers that flank the V6 region of rRNA genes according to Sogin *et al*. [8]. We assessed the product quality using a BioAnalyzer Agilent DNA 1000 LabChip following the manufacturer's instructions. Three reactions were combined, purified, and concentrated using the MinElute PCR Purification Kit (Qiagen). The final amplicon library was sequenced independently by 454 Life Sciences and our own lab. Both labs used the Roche Genome Sequencer 20 (GS20) according to the manufacturer's specifications [2]. The original GS20 output files as text are available in Additional data files 3-5.

### Error rate calculations

We combined the data from both sequencing runs for a total of 340,150 reads (226,150 and 114,000), with an average read length of 94.5 nucleotides and a total of 32,816,656 bases. These sequences are available in fasta format in Additional data file 2. To determine the reference sequence source of each pyrosequencing read, we ran a separate multiple sequence alignment of each individual read against the 43 reference sequences using MUSCLE [16] (default options plus maxiters 2, diags). We calculated the number of sequence differences between each read and the reference sequences to determine the reference sequence to which each read mapped most closely. All subsequent error calculations are based on comparing reads to their assigned reference sequence.

The overall error rate is the number of errors in a read divided by the length of sequence. Specifically, we calculated errors in several ways. In all methods, each base mismatch or N in the test sequence counts as an error, and a terminal gap caused by a GS20 read terminating before the end of the reference does not count as an error. In the first and second methods each base of an insertion or deletion counts as one error. In the third method, insertions or deletions are counted by homopolymer runs. If a TAAA is inserted, it is counted as two insertions, one single-T and one multi-A insertion. The denominator for the first method was the read length. The denominator for the second and third methods was the length of reference sequence minus any discounted terminal gaps. All error rate calculations produced essentially the same results. We report error rates using all base errors (not by homopolymer run) divided by the expected length (reference sequence length minus terminal gaps).

The error rates were calculated for each sequence in a pairwise comparison of the pyrosequencing read and the reference sequence to which it was assigned. We used the Needleman-Wunsch algorithm [19] for these pair-wise alignments because it selects for the best possible alignment given its run parameters. Using a set of 100 sequences and a matrix of Needleman-Wunsch run parameter combinations, we found that a gap opening penalty of 5.75 and a gap extension penalty of 2.75 minimized the calculated error rate. Error rates, reference sequences and read sequences were imported into a MySQL database for storage and analysis.

## Additional data files

The following additional data are available with the online version of this paper. Additional data file 1 is a fasta file of the 43 known sequences used. Additional data file 2 is a gzip-compressed fasta file of the sequences output by the GS20. These sequences correspond to those included in Additional data files 3, 4, 5 but include only the final sequence information. Additional data files 3, 4, 5 are three compressed text files representing the text translations of the original GS20 binary output (sff) files for all of the sequencing used in the analysis, including sequence, flowgram and other run information. GS20 data are reported by region of the PicoTiterPlate™; we sequenced three plate regions.

## Supplementary Material

Additional data file 1 The 43 known sequences used Click here for file

Additional data file 2 These sequences correspond to those included in Additional data files 3-5 but include only the final sequence information in fasta format. Click here for file

Additional data file 3 Text translation of the original GS20 binary output (sff) file for the first of three PicoTiterPlate™ regions used in the analysis. Click here for file

Additional data file 4 Text translation of the original GS20 binary output (sff) file for the second of three PicoTiterPlate™ regions used in the analysis. Click here for file

Additional data file 5 Text translation of the original GS20 binary output (sff) file for the third of three PicoTiterPlate™ regions used in the analysis. Click here for file
